# The relationship between social anxiety and problematic smartphone use: the serial mediation model of perceived social support and smartphone use frequency

**DOI:** 10.3389/fpubh.2026.1865846

**Published:** 2026-07-17

**Authors:** Chen Liu, Zhiqiang Hao

**Affiliations:** 1 Tianjin Sino-German University of Applied Sciences, Tianjin, China; 2 Office of International Affairs, Nankai University, Tianjin, China

**Keywords:** college students, perceived social support, problematic smartphone use, serial mediation model, smartphone use frequency, social anxiety

## Abstract

**Objectives:**

Parents and educators have long been concerned about the development and consequences of problematic smartphone use (PSU) among students, especially college students who have more free time at their disposal. Few studies have explored the correlational association between social anxiety and PSU via potential statistical linkage pathways. This study explored correlational associations between social anxiety and PSU and tested a serial statistical mediation model including perceived social support and smartphone use frequency.

**Methods:**

An online survey was conducted among college students from a university in China using the Social Interaction Anxiety Scale, the Perceived Social Support Scale, the Smartphone Use Frequency Scale, and the Smartphone Addiction Scale-Short Version. A total of 597 valid questionnaires were obtained (72.86% male). The serial mediation analysis was performed using Hayes’ PROCESS macro (Model 6).

**Results:**

The results indicated that social anxiety and smartphone use frequency were positively correlated with PSU; perceived social support was negatively correlated with both social anxiety and PSU. All correlation coefficients reached a statistically significant level (all *ps* < 0.05). Perceived social support and smartphone use frequency showed statistically significant indirect linking effects in the correlational model of social anxiety and PSU.

**Conclusion:**

In the correlational model, perceived social support and smartphone use frequency exhibited a statistically significant serial indirect association pattern linking social anxiety and PSU; these correlational findings provide a reference for PSU prevention research.

## Introduction

As an old Chinese saying goes, “Too much of a good thing is good for nothing.” Smartphones are indispensable, yet their problematic use carries significant negative consequences. The 57th Statistical Report on China’s Internet Development released by the China Internet Network Information Center (CNNIC) showed that China had 1.125 billion internet users by the end of 2025, with 99.6% accessing the internet via smartphones, making mobile devices the primary online tool nationwide (1). Mobile phones have become the most commonly used tool for college students in the new era to engage in online activities. Problematic smartphone use (PSU) is generally defined as excessive smartphone use that leads to impairments in social or occupational functioning, including symptoms seen in dependency and addictive disorders such as withdrawal and tolerance (2). PSU can severely affect students’ physical and mental health as well as social adaptation, such as lower academic performance and inefficient learning methods (3), depression (4), and anxiety (5). Given these negative consequences, it is necessary to explore correlational predictors and potential statistical association pathways of PSU.

The Interaction of Person-Affect-Cognition-Execution (I-PACE) model posits that addictive behaviors are the result of interactions between vulnerability variables (core characteristics involved in the addiction process), emotional and cognitive responses to specific stimuli, and executive functions (such as inhibitory control and decision-making) (6, 7). Psychopathological factors, especially depression and social anxiety, have been repeatedly reported in various studies on behavioral addictions (8, 9).

Furthermore, evidence has revealed a link between media addiction and dysfunctional psychological traits and cognition, particularly in the social domain—more precisely, social anxiety (10). This provides an important theoretical basis for exploring the potential relationship between social anxiety and PSU.

Notably, as a crucial psychological protective factor, social support has been proven to buffer the adverse effects of negative emotions on behaviors (11). However, the impact of individuals’ perceived level of social support on PSU remains inconclusive. Meanwhile, smartphone use frequency itself may also play a certain role in the development of PSU. Elhai et al. (12) conceptualized anxiety as a risk factor for PSU by mediating anxiety-related transdiagnostic factors such as increased smartphone use. Based on this, the present study aims to systematically examine the relationship between social anxiety and PSU, and further examine correlational linkages of perceived social support (as a key variable in emotional regulation) and smartphone use frequency within the social anxiety–PSU association.

### Social anxiety and PSU

Previous studies on the predictive factors of PSU have repeatedly investigated and confirmed anxiety as a common psychophysiological factor (10, 12, 13).

Cross-cultural meta-analytic evidence robustly supports this correlation: Ran et al. (14) conducted a three-level meta-analysis covering global samples and confirmed a stable positive association between social anxiety and problematic mobile phone addiction across diverse cultural groups. However, research focusing on correlational links between social anxiety and PSU remains relatively limited. Social anxiety refers to an individual’s fear of potential negative evaluations in social environments, where individuals often experience high levels of anxiety and stress (15). Excessive concern about social relationships can lead socially anxious individuals to avoid social or public occasions (16, 17).

Smartphones, characterized by portability and ease of use, can eliminate the embarrassment of face-to-face communication with others (17) and create a relatively safe space for socially anxious individuals to maintain contact with others.

Additionally, seeking interpersonal relationships in online environments can compensate for the satisfaction deficit caused by the lack of face-to-face interpersonal relationships among socially anxious individuals (16, 18). The I-PACE model suggests that when facing negative emotions, people may experience impulses to regulate these emotions, and PSU may serve as a dysfunctional way to cope with such negative emotions (6). Kardefelt-Winther (19) compensatory internet use theory (CIUT) assumes that negative emotions encourage some people to overuse technology, eventually leading to problematic behaviors, namely, compensation behaviors. PSU can be conceptualized as a compensatory behavior to regulate social anxiety (20).

Therefore, based on theoretical and empirical foundations, we propose:

*Hypothesis 1 (H1)*: Social anxiety is expected to show a positive concurrent correlation with PSU.

### The mediating role of perceived social support in the relationship between social anxiety and PSU

Social support is a crucial factor in relationships (21). It can be conceptualized as perceived social support—defined as the belief that help is available when needed (22). We conceptualize social support as perceived social support because social anxiety is directly associated with it (23). Studies have shown that social anxiety is linked to lower perceived social support (23). This is understandable: Socially anxious individuals tend to be inward-focused (24) and exhibit an interpretive bias, perceiving ambiguous situations as negative and moderately negative situations as catastrophic (25). They often perceive negative evaluations from social environments, which easily leads to feelings of social exclusion (24, 25). Perceived social support can adjust an individual’s perception of stressful events, as they believe they have sufficient resources to resist threats (26), making them less likely to experience anxiety following stressors (27).

Furthermore, perceived social support may act as a protective factor against PSU (28, 29), with a negative correlation between perceived social support and PSU (30). This can be interpreted as higher perceived social support reducing the likelihood of problematic smartphone behaviors (31). The stress-buffering hypothesis states that positive interpersonal factors can buffer the psychological and behavioral impacts of stress (32).

Research has shown that individuals with high perceived social support can adapt well to both low-stress and high-stress environments and have a lower risk of addiction (33, 34). Perceived social support is associated with fewer addictive behaviors (35) and can alleviate PSU (36). Emotional and social support obtained from friends and social interactions can fully compensate for loneliness, dispel negative emotions, and help individuals avoid developing addictive behaviors (37). Based on the above analysis, we propose:

*Hypothesis 2 (H2)*: Social anxiety is expected to correlate negatively with perceived social support, and perceived social support is expected to correlate negatively with PSU; perceived social support is hypothesized to form an indirect correlational pathway between social anxiety and PSU.

### The mediating role of smartphone use frequency in the relationship between social anxiety and PSU

CIUT (19) can be used to understand the factors leading to increased smartphone use and problematic use. This theory suggests that people use or overuse internet technology as a means to alleviate negative emotions, but excessive use may ultimately result in problematic behaviors. Online, individuals with social anxiety can obtain easier and more satisfactory social interaction because their anxiety level is reduced (20). Social interaction through smartphones replaces face-to-face contact that makes individuals more anxious, and the sense of anonymity can reduce individuals’ concerns about their appearance (20, 38).

It is evident that individuals’ negative emotions, such as social anxiety, may also be closely related to smartphone use frequency—they can use smartphones to alleviate social anxiety and the resulting negative emotions. Meanwhile, existing studies have confirmed the relationship between smartphone use frequency and PSU (20, 39), with higher smartphone use frequency increasing the likelihood of PSU (40).

When facing stressful events, individuals with higher levels of perceived social support tend to perceive that they can access more social support, thus having sufficient confidence to cope with difficulties. However, the “Poor-get-Richer model” suggests that individuals with limited perceived social support may utilize new communication opportunities to establish relationships and gain support online (41, 42), and they may have stronger intentions or motivations to benefit from the internet (43). Research has shown that college students with low perceived social support have a stronger motivation to use the internet to relieve stress and seek more online social support in stressful situations (44). Given that smartphones offer efficient and convenient functionality for communicating with others anytime and anywhere, smartphone use frequency may consequently increase. Based on the above analysis, we propose:

*Hypothesis 3 (H3)*: Smartphone use frequency is expected to form an indirect correlational pathway between social anxiety and PSU; perceived social support is expected to show a positive concurrent correlation with smartphone use frequency.

### The present study

**Figure 1:**
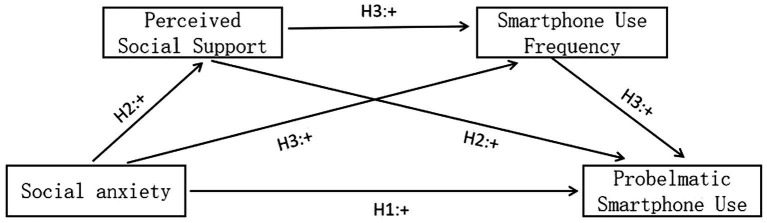
Theoretical chain mediation model.

In line with the Compensatory Internet Use Theory (CIUT), overuse of smartphones among socially anxious people acts as a way to relieve negative emotions, which in turn gives rise to PSU—framed as a compensatory response for regulating social anxiety. Furthermore, the studies discussed above have identified associations between social anxiety, smartphone use frequency, perceived social support, and PSU. It is also hypothesized that smartphone use frequency and perceived social support mediate the relationship between social anxiety and PSU, indicating that social anxiety has direct and indirect correlational links with PSU within the tested statistical model. This study constructs a serial statistical mediation model to analyze correlational pathways connecting social anxiety and PSU via perceived social support and smartphone use frequency. The theoretical model is illustrated in Figure 1.

## Methods

### Participants

A total of 630 college students were recruited via convenience sampling from a university in Tianjin. Questionnaires were distributed online, and 33 invalid responses due to abnormal completion time and repetitive answers were excluded. Ultimately, 597 valid questionnaires were retained, representing an effective response rate of 94.76%. The sample included 435 males and 162 females, with participants aged 18–23 years (Mean = 19.45, SD = 1.17).

### Instruments

#### Smartphone addiction scale-short version (SAS-SV)

PSU was measured using the 10-item Smartphone Addiction Scale-Short Version (SAS-SV) developed by Kwon and colleagues (45). Sample items include “I delay my original learning plans or tasks due to using my mobile phone” and “I feel anxious when my mobile phone is not around.” Responses were rated on a 6-point Likert scale ranging from 1 (“strongly disagree”) to 6 (“fully agree”). Higher scores indicate a higher level of PSU. The Cronbach’s *α* coefficient of the scale is 0.911, and in the current study, it was 0.922. The scale is reliable and valid (20, 46).

#### Social interaction anxiety scale (SIAS)

The Social Interaction Anxiety Scale (SIAS) compiled by Mattick et al. (47) and revised by Ye et al. (48) was used. The scale consists of 19 items, all rated on a 5-point Likert scale (1 = “completely inconsistent” to 5 = “completely consistent”), with items 8 and 10 scored in reverse. It assesses anxiety and fear in interpersonal interaction scenarios, with higher scores indicating higher levels of social anxiety. The Cronbach’s *α* coefficient of the revised Chinese version is 0.874, and in the current study, it was 0.953. The scale has established psychometric properties (20, 49).

#### Perceived social support scale (PSSS)

The Perceived Social Support Scale (PSSS) developed by Zimet et al. (50) and adapted by Wang et al. (51) emphasizes individuals’ self-understanding and self-perception of social support. The scale includes 12 items divided into 3 dimensions, measuring the level of support individuals perceive from various sources (e.g., family, friends, and others), with the total score reflecting the overall perceived social support. The internal consistency coefficients of the subscales are 0.87, 0.85, and 0.91, respectively, and the Cronbach’s *α* coefficient of the full scale is 0.88.

Responses were rated on a 7-point Likert scale (1 = “strongly disagree” to 7 = “strongly agree”). In the current study, the Cronbach’s α coefficient of the scale was 0.971. The scale is reliable and valid (52).

#### Smartphone use frequency scale (SUF)

The Smartphone Use Frequency Scale (SUF) developed by Elhai et al. (38) was used. This scale includes 12 items to assess the frequency of using specific smartphone functions, such as “voice/video calls (making and receiving),” “short messages/instant messages (sending and receiving),” “emails (sending and receiving),” and “social media (WeChat, Weibo, etc.).” Responses were rated on a 6-point Likert scale (1 = “very rarely” to 6 = “very frequently”). In the current study, the Cronbach’s *α* coefficient of the scale was 0.842. The scale is reliable and valid (39).

### Data analysis

Data analysis was conducted using SPSS Statistics v.19 and the SPSS PROCESS macro (Model 6) v.3.4 by Hayes (53). The significance of the model effects was determined using bootstrap confidence intervals (CIs) based on 5,000 random samples. If 0 was not contained in the interval, the results were considered statistically significant.

## Results

### Common method variance test

Harman’s single-factor test was conducted to examine potential common method bias in this study (54). The results revealed seven factors with eigenvalues >1. The first common factor explained 29.22% of the total variance, which was below the critical threshold of 50%. As suggested by Podsakoff and Organ (55), unrotated exploratory factor analysis yielding a single-factor variance explanation of <50% indicates negligible common method bias. Therefore, no severe common method variance was present in the current study.

### Descriptive statistics and correlation analysis of variables

Descriptive statistical analysis revealed that among the 597 college students, the scores of PSU ranged from 10 to 60 (Mean = 29.31, SD = 10.81), social anxiety scores ranged from 19 to 91 (Mean = 40.63, SD = 15.13), perceived social support scores ranged from 12 to 84 (Mean = 56.54, SD = 14.98), and smartphone use frequency scores ranged from 12 to 72 (Mean = 42.88, SD = 9.59). Correlation analysis indicated that all variables were significantly correlated with each other (*ps* < 0.05). The correlation matrix, means, and standard deviations of the variables are shown in Table 1. Correlation results confirmed the hypothesized positive concurrent association between social anxiety and PSU, verifying Hypothesis 1.

**Table 1:** Descriptive statistical results of variables and Pearson correlations (*N* = 597).

Variables	Mean	SD	1	2	3	4
1. Problematic smartphone use (PSU)	29.31	10.81	1			
2. Social anxiety	40.63	15.13	0.39***	1		
3. Perceived social support	56.54	14.98	−0.15***	−0.30***	1	
4. Smartphone use frequency	42.88	9.59	0.47***	0.10*	0.16***	1

SD, standard deviation; *p < 0.05, ***p < 0.001.

### Test of the chain mediation effect of perceived social support and smartphone use frequency

Using the SPSS macro PROCESS program developed by Hayes (53), we tested the mediating effects of perceived social support and smartphone use frequency on the relationship between social interaction anxiety and PSU, while controlling for gender and age. Specifically, Model 6 of PROCESS v3.4 was adopted, and the results are presented in Tables 2, 3.

**Table 2:** Regression results for the serial mediation model.

Result variables	Predictive variable	*R*	*R^2^*	*F*	*β*	*t*	*p*
Perceived social support	Social anxiety	0.3073	0.0945	20.62	−0.3015	−7.79	< 0.001
Smartphone use frequency	Social anxiety				0.0992	3.72	< 0.001
	Perceived social support	0.2231	0.0498	7.75	0.1287	4.78	< 0.001
Problematic smartphone use (PSU)	Social anxiety				0.2146	8.62	< 0.001
	Perceived social support	0.6038	0.3646	67.83	−0.0979	−3.86	< 0.001
	Smartphone use frequency				0.5155	13.58	< 0.001

**Table 3:** Analysis of the mediating effect between perceived social support and smartphone use frequency.

Indirect effect	Effect value	Boot SE	95% CI		Relative mediating effect
			LLCI	ULCI	
Total Effect	0.2753	0.0269	0.2226	0.3280	100%
Direct Effect	0.2146	0.0249	0.1657	0.2636	77.95%
Total Indirect Effect	0.0606	0.0165	0.0295	0.0951	22.01%
Social Anxiety → Perceived Social Support → Problematic Smartphone Use (PSU)	0.0295	0.0088	0.0132	0.0475	10.72%
Social Anxiety → Smartphone Use Frequency → Problematic Smartphone Use (PSU)	0.0511	0.0173	0.0177	0.0866	18.56%
Social Anxiety → Perceived Social Support → Smartphone Use Frequency → Problematic Smartphone Use (PSU)	−0.0200	0.0064	−0.0333	−0.0079	−7.26%

As shown in Table 3, the PROCESS program combined with the bootstrap method was used to test the significance of the aforementioned mediating effects. The results revealed three significant mediating chains: first, social anxiety → perceived social support → PSU; second, social anxiety → smartphone use frequency → PSU; third, social anxiety → perceived social support → smartphone use frequency → PSU. The confidence intervals of the above three indirect effects did not include 0, indicating that all indirect effects reached a significant level.

**Figure 2:**
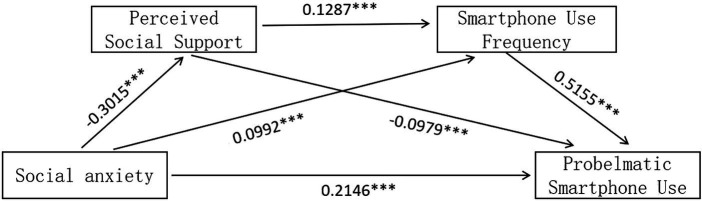
Results of the final chain mediation model. ***p < 0.001.

As shown in Figure 2, Social anxiety was significantly negatively correlated with perceived social support (*β* = −0.3015, *p <* 0.001), significantly correlated with smartphone use frequency (*β* = 0.0992, *p* = 0.0002), and significantly positively correlated with PSU (*β* = 0.2146, *p <* 0.001). Perceived social support was significantly positively correlated with smartphone use frequency (*β* = 0.1287, *p <* 0.001) and significantly negatively correlated with PSU (*β* = −0.0979, *p* = 0.0001).

Smartphone use frequency was significantly positively correlated with PSU (*β* = 0.5155, *p <* 0.001). Correlation analyses revealed higher social anxiety correlated negatively with perceived social support and positively with PSU, matching Hypothesis 2 via a significant indirect statistical path. Smartphone use frequency also served as another indirect statistical path between social anxiety and PSU, and perceived social support was positively correlated with smartphone use frequency, consistent with Hypothesis 3. The two variables together formed a significant serial indirect association in the tested statistical model.

## Discussion

Previous studies on the association between social anxiety and PSU remain relatively limited. Starting from individuals’ social anxiety issues, this study explores correlational relationships of PSU and provides tentative correlational evidence for follow-up research. There was a significant positive correlation between social anxiety and PSU (*β* = 0.39, *p <* 0.001), which verified H1. This result was consistent with previous work that identified relationships between social anxiety and PSU (6, 56). To avoid social interactions that may lead to negative evaluations, socially anxious individuals need alternative ways to replace face-to-face social behaviors to overcome loneliness and meet their normal needs for interpersonal relationships. As an accessible, easy-to-operate tool that enables communication with others anytime and anywhere, smartphones have become a way for socially anxious individuals to satisfy their desire for interpersonal connections, which is associated with excessive smartphone use and PSU. Studies have also noted that socially anxious individuals may consume non-social content to fill their time, gain satisfaction, avoid problems, and compensate for the lack of face-to-face communication (10). Regardless of the purpose, socially anxious individuals are more prone to excessive use of mobile devices, ultimately increasing the likelihood of PSU.

### The mediating effect of perceived social support and smartphone use frequency

As shown in Table 2, social anxiety was significantly and negatively associated with perceived social support (*β* = −0.3015, *p <* 0.001), and perceived social support was significantly and negatively associated with PSU (*β* = −0.0979, *p* = 0.0001). These results indicate that perceived social support negatively correlates with social anxiety and PSU, and that perceived social support plays a mediating role, verifying H2. The results indicate that higher levels of social anxiety are associated with lower levels of perceived social support and higher levels of PSU. The results were consistent with those of previous studies (23, 56). Sun et al. (57) found that perceived friend support moderated the association between social anxiety and PSU.

The social support buffering hypothesis suggests that perceived social support can buffer individuals from the negative impacts of certain risk factors (32). Davis’ cognitive-behavioral model also points out that factors contributing to problematic internet use include a lack of social support. When college students fail to establish effective social support, they may seek external support through media to alleviate the resulting negative emotions, ultimately leading to PSU (58). It is evident that individuals with higher levels of perceived social support may be less affected by social anxiety, thereby buffering the potential for problematic behaviors in anxious emotional states to a certain extent.

As shown in Table 2, social anxiety was significantly and positively associated with smartphone use frequency (*β* = 0.0992, *p* = 0.0002), and smartphone use frequency was significantly and positively associated with PSU (*β* = 0.5155, *p <* 0.001). The results also indicate that smartphone use frequency positively correlates with social anxiety and PSU, and that smartphone use frequency plays a mediating role, verifying H3. The findings indicate that social anxiety is closely related to smartphone use frequency, and smartphone use frequency can significantly positively predict PSU. This is consistent with the CIUT (19), which suggests that people use or overuse internet technology to alleviate negative emotions, and also supports the I-PACE model (6, 7), which links dysfunctional psychological traits to behavioral addictions.

### The serial mediating effect of perceived social support and smartphone use frequency

As shown in Table 2, perceived social support was significantly and positively associated with smartphone use frequency (*β* = 0.1287, *p <* 0.001), social anxiety correlated positively with smartphone use frequency (*β* = 0.0992, *p* = 0.0002), and smartphone use frequency correlated positively with PSU (*β* = 0.5155, *p <* 0.001). These findings demonstrate that perceived social support and smartphone use frequency exert a serial mediating effect between social anxiety and PSU, verifying H3.

The current study reveals the serial indirect association of perceived social support and smartphone use frequency between social anxiety and individuals’ PSU, providing new insights for the prevention and management of problematic behaviors. Notably, two competing pathways coexist in this model: the pathway through perceived social support shows a negative trend, while the pathway via smartphone use frequency presents a positive trend. These opposite associations reflect different psychological mechanisms rather than contradictory results, and the positive pathway plays a dominant role in the overall relationship. First, social anxiety is positively correlated with the severity of individuals’ problematic smartphone behaviors.

Compared with issues such as depression and test anxiety that often receive attention from families, society, and schools, social anxiety, as a common individual anxiety phenomenon, is easily overlooked, potentially leading to negative consequences.

Therefore, strategies to address problematic behaviors can start with psychological factors. Meanwhile, it is recommended that families and society of individuals with PSU provide more social support, focusing on cognitive and emotional regulation. Second, considering the indirect link of smartphone use frequency between social anxiety and PSU, parents or school teachers should pay attention to whether students with increased smartphone use frequency are experiencing negative emotions. Of course, given that smartphones are currently an important tool for learning, daily life, and work, directly prohibiting their use is inappropriate; instead, it is necessary to establish healthy usage habits based on actual circumstances.

This study has several limitations. First, the cross-sectional data collected do not allow inferences about causal relationships. Any implied causal interpretation of observed indirect effects should be avoided, and longitudinal or experimental designs are required for future causal inference. Second, the research data are derived from self-reports, which may be affected by social desirability, recall, and estimation biases.

In particular, self-assessments of smartphone use frequency may be influenced by time distortion, leading to cognitive biases. Future research can use longitudinal or experimental designs to clarify the directionality of the results and overcome limitations associated with self-reports. Third, the sample was recruited from a single university via convenience sampling and was dominated by male students, restricting the generalizability of the findings to broader college student populations. Future research will adopt multi-institution sampling and gender-stratified sensitivity analysis. Finally, future studies can continue to explore other mediating and moderating factors influencing PSU to enrich the research on the mechanisms underlying PSU.

As an investigation into the occurrence of PSU and its psychosocial predictors, this study has important theoretical and practical value. It helps to better understand how social anxiety increases the risk of PSU, as well as the importance of alleviating negative emotions through appropriate offline social support activities and obtaining social support from smartphones. Given the convenience sampling and cross-sectional design, the results should be interpreted with caution.

## Conclusion

Perceived social support and smartphone use frequency formed a statistically significant serial indirect correlational linkage between social anxiety and PSU. Perceived social support showed a significant positive concurrent correlation with smartphone use frequency. Our findings provide preliminary empirical reference for PSU prevention research. Future research can continue to conduct in-depth research on the mechanisms underlying the development of smartphone problems.

## Data Availability

The datasets presented in this study can be found in online repositories. The names of the repository/repositories and accession number(s) can be found at: https://www.scidb.cn/s/yaEJVn.
